# Design and application of a novel 3D printing digital navigation template for cubitus varus deformity in children

**DOI:** 10.3389/fped.2024.1342980

**Published:** 2024-08-07

**Authors:** Ming Zou, Youzhi He, Yuxia Xu, Qiang Shi, Hao Zeng

**Affiliations:** ^1^ Department of Sport Medicine, The Affiliated Changsha Central Hospital, Hengyang Medical School, University of South China, Changsha, China; ^2^ Department of Spine Surgery Zone 2, The Affiliated Changsha Central Hospital, Hengyang Medical School, University of South China, Changsha, China

**Keywords:** 3D printing technology, digital navigation template, cubitus varus deformity, children, template

## Abstract

**Background:**

This study was aimed to assess the feasibility and efficacy of 3D printing digital template for treatment of cubitus varus deformity.

**Methods:**

32 patients who underwent lateral closing osteotomy were evaluated between January 2018 and January 2020 in this retrospective study. Navigation templates were used in 17 cases, while conventional surgery in 15 cases. The carrying angles before and after surgery, operation time and elbow joint function were compared.

**Results:**

Navigation templates matched well with the anatomical markers of the lateral humerus. More accurate osteotomy degrees, shorter operation time and less radiation exposure were achieved in the navigation template group (*p* < 0.05). At the last follow-up time, significant difference was found based on the Bellemore criteria (*p* = 0.0288).

**Conclusions:**

The novel navigation template can shorten operation time, improve the lateral closing osteotomy accuracy and improve postoperative elbow joint function.

## Background

Supracondylar humerus fractures are very common in children, while cubitus varus deformity may occur if these patients are not treated well (1, 2). It is considered that cubitus varus deformity belongs to a three-dimensional (3D) deformity, which includes varus deformity in the coronal plane, an overextension deformity in the sagittal plane, and an internal rotation deformity in the horizontal plane (3, 4). Lateral closing-wedge osteotomy is always performed by most orthopedic surgeons for treatment of cubitus varus deformity, which demonstrates more effective than dome osteotomy or varus osteotomy (5, 6). However, accurate performance and positioning of lateral closing-wedge osteotomy during surgery are crucial to ensure ideal correction effects.

Currently, patient specific guides for navigation, osteotomies, screw/wire positioning have been used for decades. Over the last decade, 3D printing has provided an alternative manufacturing method. Based on 3D printing technology, individualized navigation templates can completely match the solid skeleton. To make the corrective surgery more accurate in children, we need navigation templates because they have individual differences. Recently, Zhang et al. designed a computer-aided design osteotomy template for treatment of cubitus varus deformity in teenagers, which was easy to use and generated highly accurate osteotomy (7). However, the navigational template was hard to be attached to the distal humerus firmly. To solve this problem, we designed a novel 3D printing digital navigation template for treatment of cubitus varus deformity and the template was fixed hard in the bone surface. This study was aimed to evaluate whether digital navigation template was simple and precise, and could achieve accurate osteotomy degree.

## Materials and methods

### Patients

From July 2016 to September 2018, thirty-two consecutive patients (17 boys and 15 girls) presenting cubitus varus deformity were reviewed. No statistical significance was found for the patient's demographic characteristics between the conventional group and navigation template group (Table 1). The inclusion criteria were as follows: (i) all cubitus varus were secondary to supracondylar fracture; (ii) carrying angles of the affected sides were more than 15° and (iii) the timing for operation was more than 12 months after the fracture occurred. All patients underwent lateral closing-wedge osteotomy. Traditional surgery for 15 cases while templates for other 17 cases. This study was approved by the institutional review board of the Affiliated Changsha Central Hospital, Hengyang Medical School. All parents gave written informed consent before participating in this study. All parents gave their written informed consent for the publication of children images.

**Table 1:** Comparison of demographic data and characteristics.

Characteristics	Conventional group (*n* = 15)	Navigation template group (*n* = 17)	*P*-value
Mean age (range), years	9.2 ± 2.6 (8–14)	9.7 ± 1.9 (8–13)	0.546
Gender, *n* (%)			0.982
Male	8 (53.3)	9 (52.9)	
Female	7 (46.7)	8 (47.1)	
Side, *n* (%)			0.755
Left	7 (46.7)	7 (41.2)	
Right	8 (53.3)	10 (58.8)	
Preoperative carrying angle (°)			
Affected side	−21.8 ± 7.3	−22.7 ± 5.9	0.404
Normal side	6.4 ± 1.8	6.8 ± 1.6	0.549

### Digital design and 3d printing navigation template preparation

Preoperatively, anteroposterior (AP) and lateral view radiographs and CT scans of ipsilateral and contralateral upper limbs were taken in all patients. The methods for digital design and 3D printing navigation template preparation were the same as our previous literature (8).

**Figure 1:**
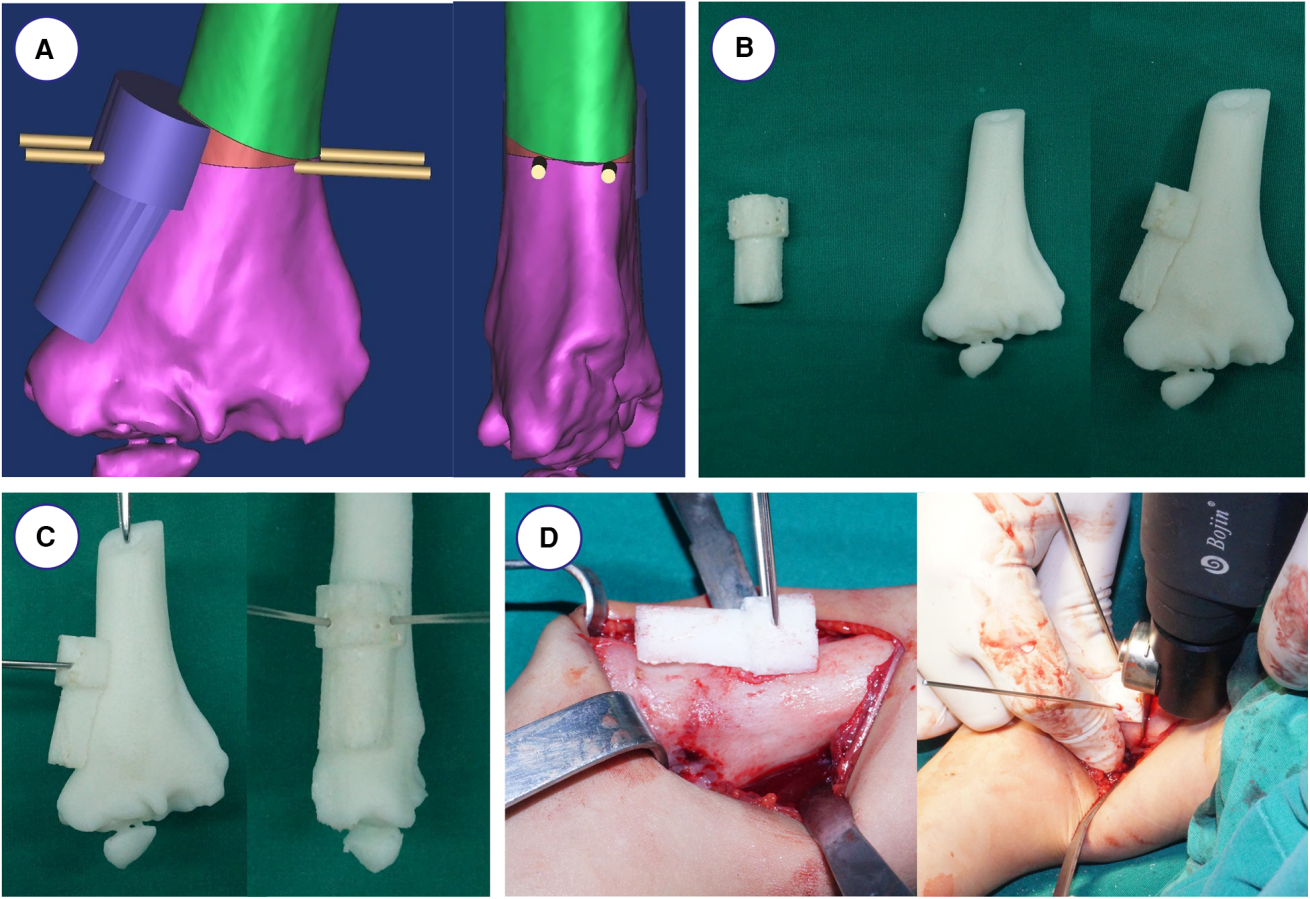
Simulated operation with 3D printing model and navigation template. (**A**) Design of osteotomy angle and plane. (**B**) The navigational template fitted 3D printing model of humerus perfectly. (**C**) The K-wires were inserted through the navigational template. (**D**) Intraoperative operation using the navigation template.

The angle of lateral closing-wedge osteotomy was calculated by computer software, and then, the angular difference at the distal end of the humerus was directly measured, at which the osteotomy was also required. The distal osteotomy plane was approximately 1.5 cm above the olecranon fossa. The Kirschner wire guide pipe was attached to the fitting surface to fix the template, which provided a mark for correcting the rotational deformity. The templates matched with the humerus well and the osteotomy operation was more convenient. Meanwhile, according to the correction amount, the proximal osteotomy plane was performed by using the Amira 3.1 software (Figure 1A). The 3D models were saved in STL format for stereolithography and the optimal osteotomy degrees were defined based on the humerus parameters using 3D printing technology (Figure 1B).

### Operation and postoperative treatment

**Figure 2:**
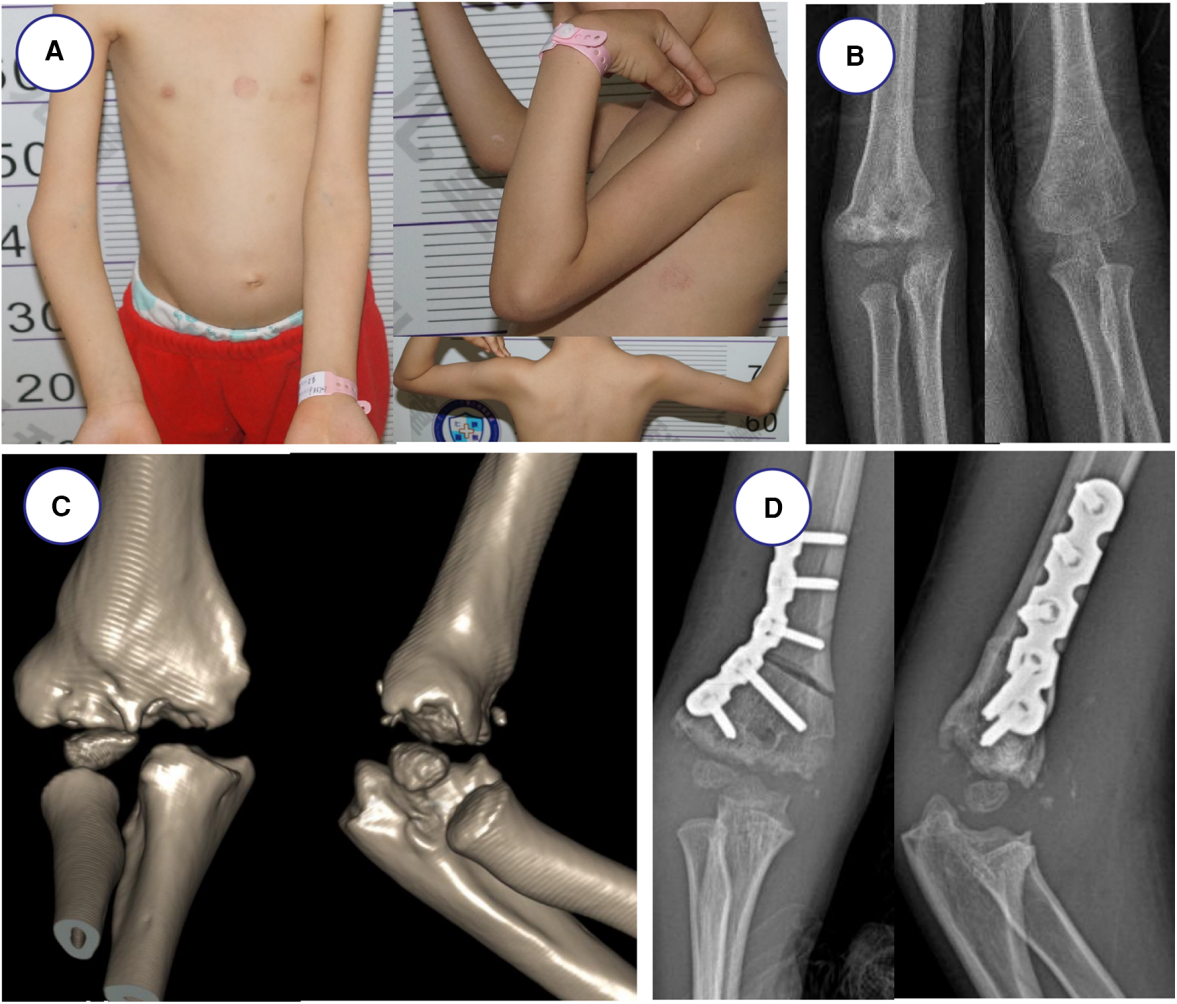
Radiographs before and after the operation in navigation template group. (**A**) Appearance showing varus deformity of the right elbow. (**B,C**) Radiographs of right elbow joint showing cubitus varus deformity before operation. (**D**) Postoperative radiograph showed anatomical correction and good appearance.

**Figure 3:**
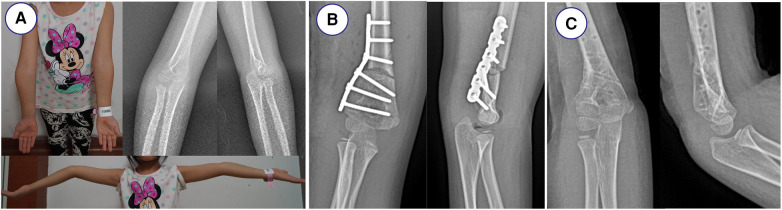
Radiographs before and after the operation in conventional group. (**A**) Appearance showing varus deformity of the right elbow. (**B**) Radiographs of right elbow joint after operation. (**C**) Radiographs of right elbow joint after removal of plate.

**Figure 4:**
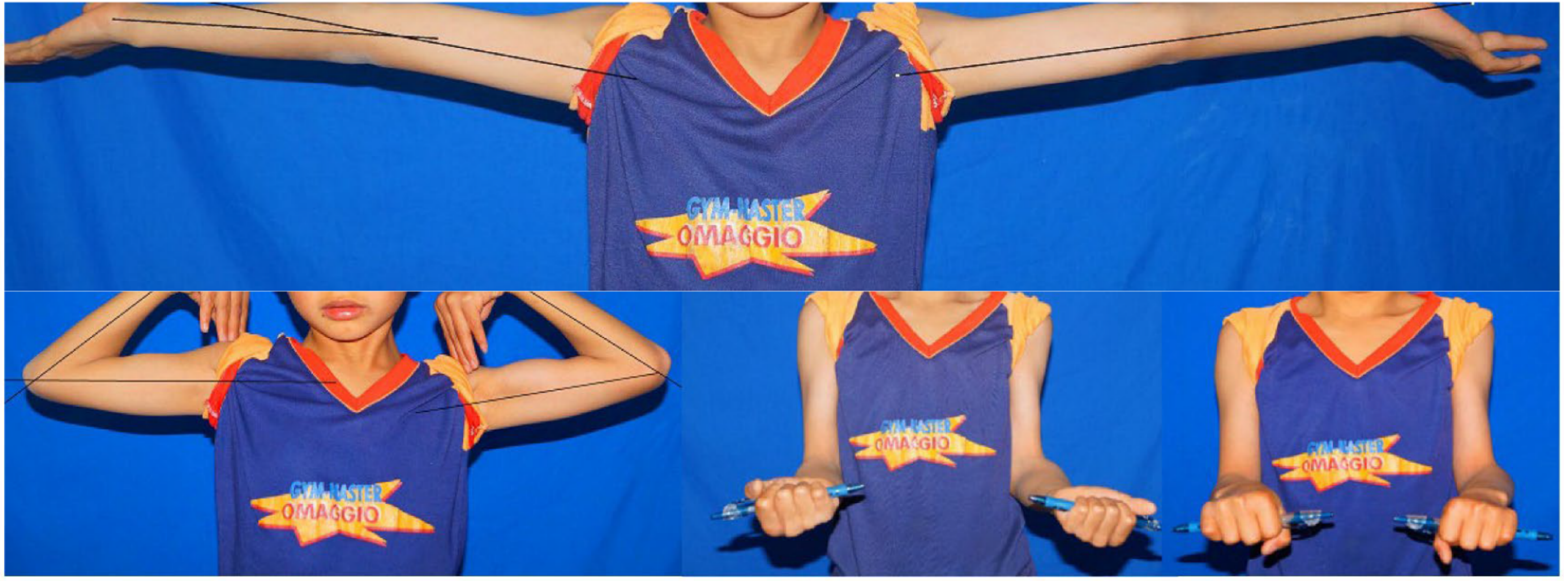
Appearance showing good correction of the deformity after surgery, with no limit to the flexion and extension of the elbow joint and rotation of the forearm.

All the surgeries were performed by one senior orthopedic surgeon. The 3D printing navigation template was sterilized and applied intraoperatively to assist lateral closing-wedge osteotomy (Figure 1C). After completing the osteotomy during the surgery, the 3D printing navigation template was removed and coincided to correct the distal internal rotation deformity (Figure 1D). The child was anesthetized and placed in a supine position, and lateral cortical bone of the distal humerus was exposed. After matching the template to the distal end of the humerus, the 3D printing navigation template was fixed with two Kirschner wires to prevent slippage. When blocking the wedge-shaped osteotomy, we remove the 3D printing navigation template and observe the affected limb. Finally, they were fixed by internal fixation with Locking Compression Plate (LCP) as preoperatively simulated (Figure 2). Meanwhile, the lateral closing-wedge osteotomy was performed according to the preoperative and intraoperative measurements in the conventional group (Figure 3). No significant difference in postoperative management procedures after lateral closing-wedge osteotomy between the two groups. The long arm plaster was fixed four weeks after surgery. Moreover, radiographs of the elbow joint were taken regularly until the region of osteotomy completely healed and then the internal fixation was removed. The function evaluations were evaluated at last follow-up time according to Bellemore criteria (Figure 4) (9).

### Statistical analysis

All measurement data were presented as means ± SD, and Student's *t*-test was used to examine the differences between groups. Chi-squared test and Fisher's exact test were applied to analyze the data of the two groups in this study. *P*-values < 0.05 were considered statistically significant.

## Results

For the 17 children with cubitus varus deformity in the 3D navigation template group, all templates matched well with the anatomical markers of the lateral humerus (by both intra-operative visual and post-operative imaging). Compared with the conventional group, there was statistical significance in radiation exposure (*p* < 0.0001) and operation time (*p* < 0.0001) in the 3D navigation template group (Table 2). Regarding the correcting degrees of carrying angle, there was statistical significance between the conventional group (25.1 ± 7.2°) and navigation template group (21.1 ± 5.3°, *p* = 0.0161).

**Table 2:** Comparison of operation data and functional outcomes between two groups.

	Conventional group (*n* = 15)	Navigation template group (*n* = 17)	*P*-value
Corrective carrying angle (°)	25.1 ± 7.2	21.1 ± 5.3	0.0161
Radiation exposure, times	8.0 ± 2.4	5.3 ± 1.7	<0.0001
Operation time, min	43.2 ± 7.5	26.7 ± 4.8	<0.0001
Maximum elbow motion (°)			
Extension	2.3 ± 3.5	1.8 ± 2.8	0.1936
Flexion	128.5 ± 4.1	126.2 ± 3.3	0.2288
Bellemore criteria			0.0288
Excellent	7 (46.7)	15 (88.2)	
Good	5 (33.3)	2 (11.8)	
Poor	3 (20)	0	

No complications, such as incision infection, nonunion, or loss of reduction, occurred in two groups. Two cases of the prominence of the lateral condyle were found in conventional group. Radiographs showed no varus, extension, or flexion deformity at the final follow-up. In the navigation template group, 15 patients obtained excellent correction and 2 patients obtained good prognosis, while in the conventional group, 7 cases were excellent, 5 cases were good, and 3 as poor. According to the Bellemore criteria, the elbow joint function between the two groups showed no significant difference (*p* = 0.0288).

## Discussion

There are some limitations in our study. First, the number of cases was small. Second, the position of the template may be affected so we must fix the template tightly. Third, the correction of rotation should be evaluated in further studies.

Until now, closing wedge osteotomy, dome osteotomy, medial opening wedge osteotomy, step-cut osteotomy, and reverse V osteotomy have been reported (10–12). Accurately controlling the correction angle in each dimension during the lateral closing wedge osteotomy is difficult (13). 3D printing individualized navigation templates are widely used in orthopedics (14–17). Based on these technologies, 3D printing navigation templates can match the solid skeleton well (Intra-operative visual and post-operative imaging), which is safe and effective for accurate control of the angle of the osteotomy (18). Recently, different 3D printing navigation templates have been designed for cubitus varus deformity and achieved wonderful clinical outcomes (7, 19, 20). The present study applied a novel 3D printing navigation template and validated the efficacy and safety. Meanwhile, we achieved more accurate osteotomy degrees, less radiation exposure, and shorter operation time (*p* < 0.05). Besides, the healing time and post-operative infection were also reduced. According to the Bellemore criteria, there was significant difference between the two groups (*p* = 0.0288).

In the present study, the navigation template had the advantages of speed, convenience, and accuracy. It was so small that it did not expand the surgical incision, which was also simple in design and convenient during surgery. Moreover, damage of the surrounding tissues and epiphyseal cartilage were hard to be found. Third, applying the navigation template can be used to develop a preoperative plan before surgery and assist surgical navigation for accurate osteotomy. A fitting surface and upper osteotomy plane were included in the 3D printing navigation template and a Kirschner wire guide pipe was attached to the fitting surface to fix the 3D printing navigation template and avoid intraoperative slippage. After removing the template, we performed the lower osteotomy and the angle between the upper and lower osteotomy surfaces was equal to the correction of the deformity angle. Lastly, the upper and lower osteotomy surfaces were completely fitted after the osteotomy, which avoided the steps on the upper and lower osteotomy surfaces. The accuracy of the 3D printed template was checked with the aid of 3D printing model before surgery, thereby eliminating the need for repeated manipulates and shortening the surgical time. Besides, fewer radiographs are needed and continual fluoroscopic monitoring can be avoided intraoperatively.

## Conclusion

In summary, the application of a novel 3D printing navigation template for children with cubitus varus deformity can reduce the operating time, achieve less radiation exposure and improve the surgical accuracy. Therefore, this method is effective and reliable.

## Data Availability

The original contributions presented in the study are included in the article/Supplementary material, further inquiries can be directed to the corresponding author.
